# The Role of Positivity on Depressive Symptoms in Women Seeking Help for Intimate Partner Violence

**DOI:** 10.3390/ijerph20227078

**Published:** 2023-11-18

**Authors:** Eleonora Crapolicchio, Vincenza Cinquegrana, Camillo Regalia

**Affiliations:** 1 Department of Psychology, Catholic University of Sacred Heart, 20123 Milan, Italy; camillo.regalia@unicatt.it; 2 Department of Psychology, University of Campania “Luigi Vanvitelli”, 81100 Caserta, Italy; vincenza.cinquegrana@unicampania.it

**Keywords:** depression, intimate partner violence, positivity, positive psychology, psychological violence, revictimization

## Abstract

Intimate partner violence (IPV) is a complex and pervasive global phenomenon. Despite extensive research on physical and sexual violence, there has been a relative lack of investigation into the detrimental and distinctive consequences of psychological violence against women. This is surprising given the profound impact it has on the psychological well-being of victims, notably in the form of depression, which is commonly observed as an outcome in cases of psychological IPV victimization. The present study analyzes the impact of psychological IPV on depressive symptoms, considering the moderating influence of personal positivity, defined as positive self-perceptions, optimistic life perspectives, and a hopeful view of the future in a sample of 171 Italian women seeking assistance from anti-violence centers in different localities of Italy. The findings show that in line with the hypothesis, the association between psychological violence and depressive symptoms is moderated by the levels of perceived positivity, even when controlling for instances of physical violence. These results and implications for interventions are discussed within the framework of existing literature on positive psychology and psychological well-being in the context of IPV.

## 1. Introduction

Intimate partner violence (IPV) is a globally recognized form of violence against women, encompassing a wide spectrum of behaviors ranging from psychological, physical, and sexual violence to threats of such acts, including coercive tactics and controlling behaviors [1,2,3]. Research has estimated that approximately one in three women worldwide (35%) has experienced at least one incident of IPV [4].

The pervasive prevalence of IPV presents not only social and economic challenges but also harmful consequences for victims. These consequences include mental health disorders such as sleep disturbances, eating disorders, emotional distress, depression, post-traumatic stress disorder (PTSD), and alcohol use disorder [4,5,6,7,8,9,10]. Victims also often experience limitations in their ability to engage in daily activities, care for themselves and their children, reduced work and academic performance, lower motivation and productivity, job loss, and career interruptions [4,11]. In many cases, IPV leads to the murder of a woman by a man, defined as “femicide”, “feminicide”, or “gender-related homicide” [12,13].

Psychological violence plays a significant role in IPV, although it is often challenging to detect for various reasons. Firstly, it is difficult to identify and intercept, even from the victims’ perspective [14,15,16,17]. Secondly, it frequently co-occurs with other forms of violence. Psychological violence, which includes “insults, belittling, constant humiliation, intimidation (e.g., destroying things), threats of harm, threats to take away children” [2] (p. 1), often accompanies physical and/or sexual aggression by intimate partners. Nonetheless, psychological violence is widely recognized as a precursor of physical and sexual violence in intimate relationships [18,19,20,21,22,23]. Cadely and colleagues (2020) [18] examined the temporal association between psychological violence and physical violence in 434 young adults, revealing that psychological aggression positively predicted subsequent physical aggression, while early physical aggression was not a significant predictor of later psychological aggression. Psychological IPV is estimated to be the most common form of IPV [24,25,26,27], with one in five women having experienced psychological violence at the hands of an intimate partner [26,27] or from men they know [28]. Some studies estimate the prevalence of psychological IPV to be around 10–20%, while others have reported rates as high as 80–90%. [25,29,30,31,32,33,34,35,36]. These variations suggest that perceptions and interpretations of this form of violence can differ across cultures and countries [15,37,38,39,40]. Furthermore, it has been demonstrated that women who have experienced physical abuse rate the health impact of psychological violence, in terms of PTSD, depression, and anxiety, as more significant and distinct than other forms of IPV, such as sexual assaults and physical attacks, which are typically considered more severe forms of IPV [32,41,42,43,44,45,46,47]. However, much of the longitudinal research has predominantly focused on IPV and depression [6,48,49,50,51,52], as previous research has indicated a natural recovery from PTSD in IPV victims with a reduction in violent episodes [53,54]. At the same time, earlier studies have revealed a significant reduction in PTSD symptoms over time in IPV survivors during their residency in shelters [53,54]. Specifically, symptoms of psychological IPV include low mood, low energy, low self-esteem, poor self-image, and a diminished ability to think clearly [10,42,55,56], akin to depressive symptoms [57,58,59,60]. Given our focus on the effects of psychological violence, we have centered our investigation on depression as the most prominent and enduring psychological effect caused by psychological IPV [61,62,63,64]. For instance, in a study of newlyweds, psychological IPV was found to predict higher levels of depression, even after controlling for physical IPV [46]. In a systematic review, Lagdon and colleagues (2014) [42] analyzed the effects of IPV victimization on mental health and demonstrated that, after accounting for other types of violence, psychological violence had a direct impact on depressive outcomes. Furthermore, some evidence suggests that depressive symptoms can increase the risk of subsequent IPV revictimization, inhibiting appropriate actions such as leaving an abusive situation [48,55,61,62,63,64,65,66]. In a study by Iverson et al. [62], reductions in depressive symptoms during treatment in 150 women survivors of IPV were associated with a decreased likelihood of IPV revictimization at a 6-month follow-up, even after controlling for recent IPV. Given these considerations, it is essential to focus on this insidious form of violence. Moreover, it is imperative to identify individual protective factors that may mitigate the negative outcomes in victims of psychological IPV to enhance personal well-being and reduce the risk of revictimization, given the vulnerability that characterizes IPV survivors with adverse outcomes as depressive symptoms.

While clinical interventions provide recommendations and guidelines on healthcare policies and provisions to address maladaptive adjustment in IPV victims [67], health and social care professionals have important knowledge gaps concerning improving the mental health of IPV victims. This involves addressing primary research concerns rather than solely focusing on the symptoms [68]. Specifically, a recent systematic review and meta-analysis [69] highlighted the limited effectiveness of psychological interventions that have been applied to treat common mental disorders in IPV victims, such as depression. It suggests the need for interventions that consider the specific impact of IPV on symptom management. For these reasons, we focus on a crucial construct in positive psychology, positivity, which encompasses not only aspects related to self-concept but also the evaluation of one’s life and expectations for the future to explore its impact on depressive symptoms [70].

### 1.1. Positivity as a Protective Factor in Depressive Symptomatology

Positivity, a construct rooted in positive psychology [71], refers to the “individual tendency to approach the various life domains including oneself positively, one’s future and past experiences” [72] (p. 277), and it is considered a key factor in explaining individual health and well-being [73,74]. According to Beck’s cognitive theory of depression, individuals affected by depression hold negative schemas about themselves, the future, and the world [75] and report a diminished experience of positivity in their lives [76,77,78]. Research in the context of positive psychotherapy has suggested that positive psychology interventions in the treatment of depression promote well-being and reduce depressive symptoms [79,80,81,82,83]. Notably, developments in the field of positive psychology indicate that higher levels of positive emotions play a crucial role in helping people cope with adversity and promoting their well-being. Additionally, positive emotions serve as a protective factor against stress and emotional vulnerability to stress [84,85,86,87]. For example, positive emotions moderated the relationship between stress and depressive symptoms in 367 military spouses during deployment, even after controlling for several demographic and deployment variables [84]. In Becker et al.’s study (Study 2) [87], positive training affected stress reactivity by consequently modifying emotional vulnerability, functioning as a buffer against stress in dysphoric and non-dysphoric individuals.

### 1.2. Positivity in the Context of Intimate Partner Violence

Research with battered women has highlighted the negative impact of IPV-related symptoms on women’s ability to utilize resources effectively and ensure long-term safety, thus increasing their risk of revictimization [48,55,61,62,63,88]. Therefore, numerous studies have focused on multiple variables in order to analyze and enhance internal coping mechanisms, such as empowerment resources (i.e., self-care, agency, self-efficacy) [89,90,91], resilience [92], decision-making power (i.e., [93]), or the economic empowerment of impoverished IPV survivors [94,95]. Additionally, studies have explored the reduction of IPV victims’ symptomatology using constructs from positive psychology to improve well-being in IPV victims, with contrasting results [41,96,97,98,99]. For example, gratitude [100,101,102,103,104] and forgiveness [105,106,107,108] are generally regarded as healthy protective factors in fostering or healing relationships. However, in particular circumstances, they can increase the risk of revictimization in the context of domestic abuse [88,99,109,110,111]. Regarding optimism, defined as a view of a future abundant with good things [112], data on its impact are mixed. A recent review [99] indicated that optimism could distort personal risk assessment and actually be unhelpful [113]. Even when they had firsthand experience with violence, optimistic participants were more likely to believe that they were at lower risk of future violence than others [114]. Berlant (2011) [115] also characterizes optimism as cruel because it can lead people to endure violence and despair if they are given the illusion of imminent improvement. Nevertheless, on the other hand, other studies found that a more realistic form of optimism, based on reality rather than wishful thinking, can help individuals be more flexible in dealing with adversity [113,116].

Given the sensitive nature of this phenomenon, we have attempted to consider a positive view of self, the future, and life [70] rather than an optimistic perspective focused on the belief that things will change for the better as the mechanism that could affect depressive symptomatology. Positivity is a pervasive positive mood that encompasses not only an individual’s expectations for the future. Previous studies [70,117] have demonstrated that positivity includes aspects related to self-esteem [118], life satisfaction [119], and optimism [120], tracing these constructs to a common factor.

To the best of our knowledge, this study represents the first attempt to analyze the role of positivity in the association between previous psychological IPV and depressive symptomatology in a sample of battered women seeking help in Italian shelters. In particular, we assume that positivity can have a moderating effect, modifying the direction or strength of associations between psychological IPV and depression [121,122]. Importantly, the hypothesis of positivity as a moderator implies that how IPV victims cope with stress can decrease or increase the consequences of traumatic events on the development of mental health disorders [123], particularly the negative effects of depressive symptoms in IPV survivors seeking help for IPV.

### 1.3. The Present Research

In line with previous research, we posit that psychological violence by the (ex)partner increases enduring psychological symptoms, such as depression. We propose that this negative outcome is moderated by positivity towards life, self, and the future as critical individual dispositions that can mitigate the severe and negative impact of psychological violence suffered by the (ex)partner. Maintaining a positive view of the self, the future, and life can act as a buffering mechanism for women, helping to mitigate the negative effects of psychological violence. Conversely, the absence of hope and trust in the future can exacerbate the negative consequences of violence, leading to increased depressive symptoms.

**Figure 1:**
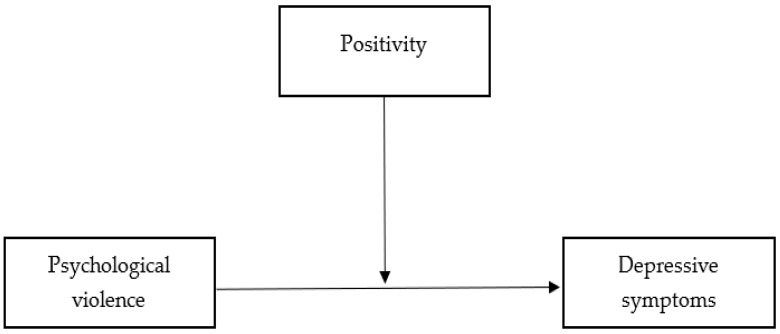
Tested model.

In summary, we hypothesize that psychological violence is positively associated with depressive symptomatology controlling for experienced physical violence(Hp1). Furthermore, we predict a moderated effect of positivity on the association between psychological violence and depression (Hp2). Specifically, we anticipate that significantly lower levels of positivity will intensify the association between psychological violence and depressive symptoms. The hypothesized model is presented in Figure 1.

## 2. Materials and Methods

### 2.1. Participants and Procedure

A convenience sample of 171 Italian women was surveyed. The women took part in an extensive study of the violence perpetrated by male partners on their intimate partners. The mean age of participants, aged between 18 and 78 years, was 44.02 years (SD = 10.17). All of them had children, and most of the women (86.5%) were separated from their partners, while 13.5% of participants were still living with him while participating in the study. Concerning the education level, 19.9% of participants had a degree, 41% of women held secondary education, 33.1% had a middle school education, and a minority of women held primary education (6%); 39.3% did not have a job (24.8% unemployed and 14.5% housewife).

The study was reviewed and approved by the Ethical Commission of the Department of Psychology, Università degli Studi della Campania “Luigi Vanvitelli”.

Participants were recruited in anti-violence shelters located in different Italian areas of northern, central, and southern Italy and in Italian emergency rooms within hospitals specialized in dealing with female victims of violence, where women survivors sought help. Although located in an Italian hospital, such an emergency room is a public anti-violence center, which also can be accessed without passing on to the emergency room.

In the first phase, researchers held meetings with professionals working in Italian anti-violence shelters and hospitals, where they explained the aim of the study. After consulting with the shelters’ directors of hospital staff, the researchers identified women suitable for participation in the study. Only Italian women who had children were eligible for the study. In cases evaluated as critical by the shelter’s internal professional team (i.e., not emotionally ready to undertake the interview for research purposes or the women made it clear to the team that they did not consent to participation in the research), the interview was not proposed.

Participants’ anonymity was guaranteed. Furthermore, women who participated in the study were informed that they could withdraw at any time from the study without explanation. After women had agreed to take part and given informed consent, academic researchers specialized in IPV conducted a face-to-face question and answer session. The questions were read, and the women’s responses were reported.

### 2.2. Measures

The measures included in the survey are presented below.

Socio-demographic information. We collected data on women’s age, nationality, level of education, presence of children, and whether they had ended the abusive relationship at the time of the interview. The results session also reports information on violence and abusive relationships.

Partner violence. We used the Conflict Tactic Scale revised Italian version—CTS [124,125] to observe the frequency and typology of intimate partner violence experienced by women in the last year. The 7-point Likert-type scale ranged from 0 (it never happened in the last year) to 6 (it happened more than 20 times in the past year). Consistent with the study’s objectives, we administered both physical and psychological subscales regarding the woman’s victimization, consisting of 20 items. The psychological violence subscale includes 8 items (e.g., “My (ex) partner insulted me or he yelled at me”; “My (ex) partner destroyed something that belonged to me”). The physical violence dimension consists of 12 items (e.g., “My (ex)partner pushed, shoved, or slapped me”; “My (ex) partner kicked me”). The Cronbach’s α was, respectively, 0.83 and 0.93.

**Figure 2:**
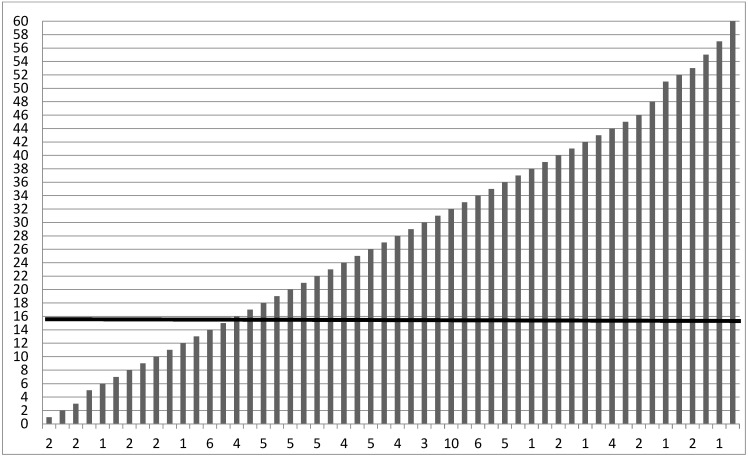
Scoring for CES-D Depression scale. The cut-off is a total score of 16 to indicate clinically significant depression. Note. Displays frequencies for the CES-D. The mean level of depressive symptoms was M = 27.05 (SD = 12.82), falling 78.9% above the cut-off score of 16 used to indicate clinically significant depression [126].

Depressive symptoms. The Italian version of the Center for Epidemiological Studies Depression (CES-D) [126,127] is a 20-item self-report scale used to measure depressive symptoms. The response rate ranged between 0 (not at all or less than once) to 3 (5–7 days or nearly every day) whether they experienced any of the symptoms listed, referring to the last two weeks. Total scores were added together, ranging from 0 to 60, with a cut-off score of 16 for the total CES-D scale for clinical significance (α = 0.79). See Figure 2 for the detailed scoring of CES-D in our sample.

Positivity. The eight-item positivity scale (POS), developed by Caprara et al. (2012) [70], was used to measure participants’ positive view of one’s self, one’s life, and one’s future, as well as their confidence in others. The scale comprises 8 items that assess different facets of positive emotions and well-being (e.g., “I feel I have many things to be proud of”; “I have great faith in the future”). The score ranged from 1 (completely disagree) to 6 (completely agree). The Cronbach’s α was, respectively, 0.84.

## 3. Results

### 3.1. Levels of Violence

Almost all of the sample suffered psychological violence (90.1%) by their (ex)partner. Specifically, most of the participants were insulted or showed (88.3%), shouted or yelled (86.5%), and their (ex)partner said something to spite them (73.7%), and most of them suffered from some kind of body shaming (80.7%) which represents severe psychological abuse (80.7%).

Furthermore, concerning physical violence, the most frequent abuses are being pushed or shoved by the (ex) partner (62.6%) or being grabbed (66.1%). About half of the sample report being slammed (49.1%) against a wall and being beaten up (55%). For detailed percentages of each abusive behavior, see Table 1.

### 3.2. Tested Model

The data collected within the database were analyzed using the SPSS statistical package (version 21.0, IBM Milano, Milan, Italy). Descriptive statistics and correlations between our variables are presented in Table 2.

As shown in Table 2, in line with the literature results, there is a co-occurrence between different forms of IPV [18,19,20,21,22,23]. The bivariate correlations showed a significant positive relationship between psychological and physical violence (B = 0.68, *p* < 0.001). Surprisingly, compared to the hypotheses, both psychological (B = 0.16, *p* < 0.05) and physical violence (B = 0.16, *p* < 0.05) positively correlated with positivity. Furthermore, the frequency of violence suffered in the last year did not correlate with levels of depression. Finally, a negative and strong relationship emerged between positive views and depressive symptomatology (B = −0.56, *p* < 0.001). In other words, women with low positive self-regard, negative views of the future, and quiet hope suffer depressive symptomatology. To answer our research questions, we ran a moderation analysis using PROCESS Macro for SPSS [128], Model 1. Specifically, we ran a regression where psychological violence was entered as the IV, positivity was the moderator, DV was depressive symptomatology, and finally, physical violence was a control variable. Results are reported in Table 3.

**Figure 3:**
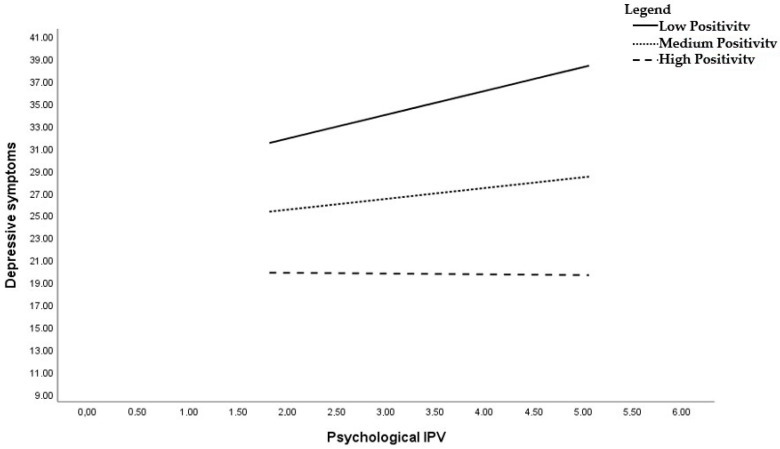
Simple slopes analysis. The effects of psychological violence on depressive symptoms moderated by positivity.

The model explained about 35% of the variance in our DV. In line with our hypotheses, psychological violence was positively related to depressive symptomatology B = 5.10, SE = 1.76, *p* = 0.004 (Hp1). Next, the association between positivity and depression symptomatology was significant, B = −3.58, SE = 1.63, *p* = 0.029. Importantly, the interaction of positivity with psychological violence was significant, B = −0.1.03, SE = 0.44, *p* = 0.019, suggesting that positivity toward self, future, and life moderates the relationship between psychological violence and depressive symptomatology, providing support for our hypothesis (Hp2). Specifically, we found that the relationship between psychological violence and depression was significant only for low levels of positivity, B = 2.15, SE = 0.76, *p* = 0.006. The results of simple slope analysis are reported in Figure 3. On the contrary, the relationship between psychological violence and depressive symptomatology was not significant when levels of positivity were high, B = −0.04, SE = 0.85, *p* = 0.956. To prevent biases in our results, we also ran our analyses controlling for physical violence, which was not significant. In addition, although IPV is a transversal phenomenon that does not have specific characteristics based on age, we performed a second moderation analysis, inserting victim age as a covariate. Such a variable was insignificant (B = 0.048, SE = 0.09, *p* = 0.595), and moderation analyses did not change regarding the direction and significance of the results.

## 4. Discussion

Intimate partner violence (IPV), in all its forms, poses a concern because of associations with multiple psychological and physical consequences, including injuries and mortality, as well as depression and PTSD [9,10]. In addition to that, the fear of living with the constant threat of violence can lead to psycho–physiological stress [52,129].

Although researchers tend to consider psychological, physical, and sexual violence as strictly interrelated in IPV, psychological violence alone can have the same far-reaching consequences for the victim as other forms of violence [41,43,130]. Prior research established that depressive symptoms are among the most common outcomes associated with psychological IPV victimization [42,43,45,46,89,131,132].

In addition, among various types of violence, psychological IPV is the most insidious form, for its diffusion, for the role of a precursor of physical and sexual violence, and for its long-lasting psychological consequences, even after the ending of the abusive relationship [88,133,134,135,136,137,138,139].

Studies also underlined that not only has psychological IPV been associated with depression or depressive symptoms, but also women experiencing depression or depressive symptoms can be at increased risk for subsequent IPV revictimization, inhibiting an appropriate action, such as leaving the situation [48,55,61,62,63,64,65,66]. In a systematic review of longitudinal studies, Devries et al. (2013) [55] revealed important evidence about the association in the reverse direction between depressive symptoms and incident IPV.

Despite this evidence, psychological violence is one of the dimensions of IPV that has received relatively less attention [140]. Furthermore, most of the previous studies mainly addressed the topic of IPV while simultaneously analyzing the impact of different forms of violence on women’s health [42,89].

The present study extends the literature on the negative effects of psychological IPV and explores how activating individual resources can mitigate them. Specifically, we focused on an individual state positively associated with psychological well-being: positivity [73,74]. Positivity comprises not only aspects related to self-concept but also the cognitive evaluation of life and expectations for the future [70], embracing the focus on IPV victims’ approach. Thus, we tested the positive vision toward self, life, and future as the moderated process that can mitigate the negative impact of IPV on depressive symptomatology (Hp3).

The results confirm our hypotheses by demonstrating the moderating effect of positivity on the psychological well-being of survivors of psychological violence. Specifically, only women who experienced psychological violence and exhibited low levels of positivity reported higher levels of depression. In other words, our findings suggest that maintaining a positive outlook on oneself and one’s future, despite the traumatic experience of violence, can mitigate the adverse impact on women’s psychological health, particularly by reducing the detrimental effects of violence on depression.

Surprisingly, we also observed a positive correlation between the level of violence and positivity. This means that as the severity of violence increased, the positivity levels of women who had experienced physical and psychological violence in the past year also increased. Several characteristics of the study’s participant sample may help explain this result. The participants were women who had largely terminated their abusive relationships and had, after years of silence, sought assistance from anti-violence centers. It is likely that the intensity and frequency of the violence they endured pushed them to make significant life changes. Seeking help and encountering professionals who could address their suffering likely instilled genuine hope for their future and boosted their self-esteem due to the achievements they had made.

Another possible interpretation relates to the potential downside of positivity. Some research indicates that optimism can be a cognitive bias [141], which leads to underestimating the harmful effects of violence and reduces their inclination to take protective actions [112,113,114]. In light of this, supplementary analyses were conducted to explore the reverse relationship between positivity and psychological violence. These analyses revealed a significant effect of positivity on both psychological IPV (B = 0.25 SE = 0.12, *p* = 0.038) and physical IPV (B = 0.27 SE = 0.13, *p* = 0.034). This suggests that positivity can also be associated with an increase in psychological and physical IPV. Consequently, the promotion of a positive attitude in women experiencing IPV should be approached cautiously by professionals.

Nonetheless, the primary finding of this study underscores that a positive outlook on the future can serve the purpose, at least in the short term or in specific phases of the cycle of violence, of reducing the anxiety and stress associated with a traumatic and uncertain situation concerning the evolution of the future or the reactions of the violent partner. Moreover, it can help diminish the depressive symptoms linked to the experiences endured. Depressive symptoms can continue to affect victims for an extended period, even years after the abusive relationship has ended. Strengthening women’s self-confidence and fostering a positive self-perception, along with a positive view of life and the future, despite the violence suffered, appears to make a significant difference. In this regard, anti-violence centers may indeed play a pivotal role in promoting well-being and rebuilding the lives of survivors of gender-based violence. Specifically, they provide a safe and confidential space where survivors can share their experiences without judgment or fear. In this direction, anti-violence center operators trained in gender-based violence and survivor-centered approaches may effectively foster a sense of trust and security, enabling women suffering from violence to express their feelings, concerns, and trauma openly and plan their future. These findings have several important implications. First, they underscore the importance of providing information to both women and professionals on how to recognize and address situations of psychological violence. Given that partner psychological violence is often subtle and difficult to detect [14,15,16,17], it is essential to educate individuals to recognize the signs, make victims aware of their situation, and highlight the adverse effects it has on women’s health. Second, it is crucial to empower women with a positive vision of the future, which may involve safeguarding them from the risk of revictimization, providing practical tools for rebuilding their lives, and offering access to psychotherapeutic support. This assumes a central role in the lives of women who have experienced violence.

Second, delving into the history and experiences of violence is fundamental for survivors. The work of anti-violence centers is essential for promoting the emancipation and self-determination of women who have been abused by their current or former partners. In addition to addressing the traumatic history of intimate partner violence (IPV), it is equally important to enhance women’s empowerment and cultivate a positive, violence-free vision of the future. Strengthening their self-perception, their outlook on life, and their vision of the future enables them to plan their lives more effectively and build individual protection that mitigates or limits the negative psychological consequences of violence. The absence of hope, trust, and a negative self-view partially characterizes depressive symptoms. Positivity can be considered the opposite of these emotional states, highlighting the protective value of this dimension. A therapeutic approach in this direction is desirable, even in the absence of clinically significant depressive symptoms, as the lack of future planning, hope, and trust may also be linked to the protracted nature of abusive relationships and the difficulty of finding safety from a violent partner.

Despite these considerations and the practical implications of the results, the study has certain potential limitations that should be considered when interpreting the findings and planning future research. Firstly, the data are cross-sectional and do not establish causal relationships. Future studies could replicate these results using a longitudinal approach to investigate the impact of positivity on the psychological well-being of IPV survivors in the short and long term, as well as on the risk of revictimization. They might involve exploring the role of positivity at different stages of the abuse history, such as before and after a definitive separation from the abuser. Additionally, research could investigate whether a positive vision about the future following IPV experiences plays a role in preserving self-esteem and reducing anxiety and uncertainty. Moreover, future studies can explore the impact of positivity not only in terms of reducing adverse psychological effects but also from a behavioral perspective, examining whether it is a necessary mechanism for breaking free from an abusive situation, i.e., envisioning a positive future beyond the challenges, or if it increases the risk of reconciliation with the abuser and, consequently, the risk of future violence.

Another limitation is that the study considered only an individual protective factor (positivity) that mitigates the negative consequences of psychological IPV. Future research could investigate the interaction of this variable with other dimensions related to the social context (e.g., social or family support) and to the abusive relationship (e.g., ongoing custody disputes, legal cases stemming from partner abuse, partner stalking behavior post-relationship termination) that may impact women’s depressive symptoms. Furthermore, the study did not collect data on the vulnerability of the victims (e.g., dependence on alcohol or drugs) even though existing literature demonstrates a bidirectional relationship with depressive symptoms [36,142]). The study employed a convenience sample due to restrictions imposed by anti-violence centers and emergency services to protect women with IPV experience. Future research should test the study hypothesis with diverse victim samples and collect information from participants regarding potential substance addictions.

## 5. Conclusions

Notwithstanding these limitations, the results of this study provide initial evidence of the beneficial role of positivity in terms of psychological health, mitigating the negative impact of violence on depressive symptoms. This study underscores the crucial role of individual resources that can serve as buffers and aid women in coping with the devastating effects of IPV on their psychological health. In conclusion, considering the high prevalence of intimate partner violence and its substantial impact on the mental health and well-being of survivors, it is crucial to develop targeted interventions aimed at reducing risk factors for revictimization, particularly in the case of depressive symptoms. Psychological violence, which is frequently encountered and less investigated, is a significant aspect of IPV that should be further studied and addressed to better support survivors and promote their long-term well-being.

## Figures and Tables

**Table 1:** Women experiencing IPV in the previous 12 months (N = 171).

	%
**Psychological abuse**	90.1
**Physical assault**	78.4
**Psychological abuse—Minor subscale**	
Insulted me or swore at me	88.3
Shouted or yelled at me	86.5
Stomped out of the house during a disagreement	50.9
Said something to spite me	73.7
**Psychological abuse—Severe subscale**	
Threatened to hit me or throw something at me	42.7
Destroyed something belonging to me	44.4
Called me fat or ugly	80.7
Accused me of being a lousy lover	51.5
**Physical assault—Minor subscale**	
Pushed or shoved me	62.6
Grabbed me	66.1
Twisted my arm or pulled my hair	53.8
Threw an object at me with intent to injure	40.9
Slapped me	48
**Physical assault—Severe subscale**	
Slammed me against a wall	49.1
Beat me up	55
Punched me with something	31.6
Choked/Strangled me	31
Kicked me	30.4
Threatened me with a knife or gun	19.9
Burned or scalded me	4.7

**Table 2:** Descriptive Statistics and Bivariate correlations (N = 171).

Variables	1	2	3	4	M	SD
1. Psychological violence	1	0.68 **	0.16 *	0.04	3.32	1.65
2. Physical violence		1	0.16 *	−0.02	1.81	1.78
3. Positivity			1	−0.56 **	3.92	1.06
4. Depressive symptoms				1	27.06	12.82

* *p* < 0.05. ** *p* < 0.01.

**Table 3:** Results of moderation analysis.

Predictors	Dependent Variable Depressive Symptomatology	95% CI
	B	
Psychological violence (a)	5.10 (1.76) **	[1.62, 8.58]
Positivity (b)	−3.58 (1.63) *	[−6.81, −0.35]
Interaction (a × b)	−1.03 (0.44) *	[−1.90, −0.17]
Physical violence (covariate)	−0.12 (0.62)	[−1.34, 1.10]

Note: * *p* < 0.05 ** *p* < 0.01.

## Data Availability

The data supporting the findings of this study are available from the corresponding author, [E.C.], upon reasonable request.
